# A Comparison of Selective Pressures in Plant X-Linked and Autosomal Genes

**DOI:** 10.3390/genes9050234

**Published:** 2018-05-03

**Authors:** Marc Krasovec, Bruno Nevado, Dmitry A. Filatov

**Affiliations:** Department of Plant Sciences, University of Oxford, South Parks Road, Oxford OX1 3RB, UK; marc.krasovec@plants.ox.ac.uk (M.K.); bruno.nevado@plants.ox.ac.uk (B.N.)

**Keywords:** X-chromosome, faster-X effect, positive selection, purifying selection, population size

## Abstract

Selection is expected to work differently in autosomal and X-linked genes because of their ploidy difference and the exposure of recessive X-linked mutations to haploid selection in males. However, it is not clear whether these expectations apply to recently evolved sex chromosomes, where many genes retain functional X- and Y-linked gametologs. We took advantage of the recently evolved sex chromosomes in the plant *Silene latifolia* and its closely related species to compare the selective pressures between hemizygous and non-hemizygous X-linked genes as well as between X-linked genes and autosomal genes. Our analysis, based on over 1000 genes, demonstrated that, similar to animals, X-linked genes in *Silene* evolve significantly faster than autosomal genes—the so-called faster-X effect. Contrary to expectations, faster-X divergence was detectable only for non-hemizygous X-linked genes. Our phylogeny-based analyses of selection revealed no evidence for faster adaptation in X-linked genes compared to autosomal genes. On the other hand, partial relaxation of purifying selection was apparent on the X-chromosome compared to the autosomes, consistent with a smaller genetic diversity in *S. latifolia* X-linked genes (π_x_ = 0.016; π_aut_ = 0.023). Thus, the faster-X divergence in *S. latifolia* appears to be a consequence of the smaller effective population size rather than of a faster adaptive evolution on the X-chromosome. We argue that this may be a general feature of “young” sex chromosomes, where the majority of X-linked genes are not hemizygous, preventing haploid selection in heterogametic sex.

## 1. Introduction

Sex chromosomes are unusual in many respects, as they are subject to sex-biased inheritance and differences in effective population size (*N*_e_), ploidy, and recombination rate between the sexes—all of which can affect the evolution of sex-linked genes. The molecular evolution of Y (or W)-linked genes is strongly influenced by the lack of recombination, which results in gradual genetic degeneration of the sex-specific chromosome [1]. On the other hand, X (or Z)-linked genes recombine in the homogametic sex, preventing degeneration. Nevertheless, X (or Z)-chromosomes do have their fair share of distinctive features [2]. In particular, X (or Z)-chromosomes spend two-thirds of the time in the homogametic sex (assuming 50:50 sex ratios), which results in gradual “feminisation” of the X-chromosome [3,4,5] and “masculinisation” of the Z-chromosome [6]. Furthermore, X (or Z)-linked genes are hemizygous in the heterogametic sex, exposing recessive mutations to selection and significantly altering fixation probabilities for mutations in X (or Z)-linked genes [7]. The expectations for fixation probabilities and evolutionary rates on the X-chromosome were developed for mammal- or Drosophila-like sex chromosomes that are fully hemizygous in the heterogametic sex [8,9]. However, it is unclear whether these expectations are applicable to species with younger sex chromosomes that retain functional X- and Y-linked gametologs, such as many (if not most) species beyond *Drosophila*, mammals, and birds [10]. 

The effectiveness of natural selection depends on several factors, such as the effective population size, the extent of dominance, and the distribution of selective coefficients [11]. Assuming equal sex ratios, the effective population size of an X-linked gene is three-quarters of that for an autosomal gene, meaning that drift is expected to fix a larger proportion of weakly deleterious mutations and a smaller proportion of weakly advantageous mutations on the X-chromosome. On the other hand, hemizygosity of X-linked genes in males may result in stronger purifying selection against recessive or partially recessive mutations in X-linked genes [7]. Furthermore, if phenotypic effects of adaptive mutations are partially recessive on average, then hemizygosity of X-linked genes in males is expected to result in faster adaptive evolution on the X-chromosome compared to autosomes [7], the so-called “faster-X” effect [8,9]. 

Faster-X evolution was reported for a wide range of organisms, including mammals, birds, and *Drosophila* [8], though there is a considerable variation depending on the group of organisms and type of sites analysed (e.g., see Figure 2 in [8]). This variation may, at least partly, be due to lumping together the effects of adaptive and purifying selection on evolutionary rates. In particular, both purifying and adaptive selection on partially recessive mutations are expected to work more efficiently in male hemizygous X-linked genes [7,12]. However, these types of selection are expected to have the opposite effects (slowing down and speeding up, respectively) on the evolutionary rate. The attempts to compare the rates of adaptation (rather than divergence) between the X-linked and autosomal genes revealed faster-X (or Z) adaptation in *Drosophila* [9,13], silkmoth [14], humans [15], and chimpanzee [16], but the evidence for other organisms is mixed (e.g., [17,18]). 

Many plants species with separate sexes (dioecious) are known to have sex chromosomes [19,20], and some of these species have been used for studies of sex chromosome evolution (e.g., [21,22,23]). No evidence for faster-X divergence of faster-X adaptation has been reported for plants yet, though the presence of related patterns—the larger-X effect and Haldane’s rule—were reported for dioecious *Silene* [24,25]. To better understand the evolution of sex-linked genes and the role of natural selection in this process, we investigated the patterns of genetic diversity, substitution rates, and natural selection in X-linked and autosomal genes of the dioecious flowering plant *Silene latifolia* (white campion) and closely related species which evolved separate sexes [26] and de novo sex chromosomes in the last 11 million years [27]. Recombination between the male *S. latifolia* sex chromosomes is suppressed outside the pseudoautosomal region (PAR; [21,28,29]), and the X- and Y-linked gametologs have accumulated up to 20% silent divergence [21,30]. The presence of relatively closely related dioecious and non-dioecious species in genus *Silene* [31] provides a rare opportunity to reveal how the evolution of separate sexes and the development of sex linkage affects changes in the substitution rates and selective pressures. Because of the recent origin of sex chromosomes in *S. latifolia*, at least half of the sex-linked genes have functional copies on both the X- and Y-chromosomes [21], allowing us to disentangle the effects of sex-linkage and hemizygosity on selective pressures and rates of molecular evolution. In this paper, we report the analyses of adaptive and purifying selection in *Silene* X-linked and autosomal genes in order to test whether the conventional faster-X arguments [7,8,9] are applicable to species with non-degenerate or partially degenerate Y-chromosomes.

## 2. Materials and Methods 

### 2.1. Plant Material

Plants for three dioecious and three non-dioecious *Silene* species were grown in the glasshouse (20 °C and 15 h lighting) from seeds collected in the wild. The dioecious plants used for this study included female *S. latifolia* (collected in Oxford, UK), female *Silene dioica* (Wales, UK), and female *Silene diclinis* (Xativa, Spain). The three non-dioecious species included *Silene vulgaris* (Oxford, UK), *Silene acaulis* (Scotland, UK) and *Silene gallica* (Bournemouth, UK). In addition to that, we used transcriptome sequence data for four *S. latifolia* females published previously [21,25,30]. 

### 2.2. Transcriptomic Data

RNA was extracted from actively growing shoots and flower buds, as described previously [21,25]. Total RNA from plant tissue was extracted using a Qiagen RNeasy Plant Mini Kit (QIAGEN, Manchester, UK), including the optional on-column DNAse digestion. Isolation of messenger RNA (mRNA), complementary DNA (cDNA) synthesis, and high-throughput sequencing were conducted according to the standard Illumina RNA-Seq procedure at the Oxford Genomics Centre of the Wellcome Trust Center for Human Genetics (WTCHG, Oxford, UK). High-throughput sequencing for each individual was conducted at WTCHG using an Illumina HiSeq2000 (Illuma INC, San Diego, CA, USA) instrument with 100-base paired-end reads. All sequence reads were submitted to the NCBI Sequence Read Archive database under project number PRJNA448567.

Quality checking of short sequence reads of each species was conducted with FastQC v0.11 [32]. Cutadapt v1.4 [33] was used to remove Illumina adaptors. Trimmomatic v0.32 [34] was used to trim leading and trailing bases with quality below 5, cropping reads if the average quality per base dropped below 15 over four consecutive bases. Reads shorter than 36 bp after trimming were discarded. The transcriptomes for each species were assembled de novo using Trinity version r20140413p1 [35] with default settings, except for the minimum assembled contig length (set to 300 bp). The putative coding sequences (CDSs) within each transcript were identified with TransDecoder [36] using default settings.

### 2.3. Orthology Inference and Identification of X-Linked and Autosomal Genes

To study the evolution of X-linked and autosomal genes in dioecious and non-dioecious species, we first identified orthologous genes across all six *Silene* species and subsequently focused on a subset of genes whose genomic location (X-linked or autosomal) could be confidently assigned.

Orthology inference was performed using the clustering and phylogenetic-based method of reference [37], following the protocol described previously [38]. Briefly, coding sequences of all species were first assigned to homologous clusters based on all-by-all BLASTn (Basic Local Alignment Search Tool) [39] searches followed by clustering with MCL (Markov clustering) [40]. These clusters of homologous sequences were aligned with MAFFT v7.123b [41], and the resulting alignments were used in RAxML v8.0.1 [42] to obtain homologous trees. Assembly and clustering errors at this stage were identified by the analysis of the resulting homologous trees, as previously described [37,38]. Orthologous genes were extracted from the homologous gene trees using the Maximum Inclusion method [37], and the sequences were realigned using PRANK v140110 [43] with the codon substitution matrix method [44]. 

The orthologous genes identified across all six *Silene* species were assigned to X-linked, autosomal, or of unknown linkage using the previously published genetic map of *S. latifolia* [21]. Homology between newly and previously identified genes [21,30] was performed with a reciprocal best blast hit, using representative *S. latifolia* sequences for each orthologous gene. In addition, X-linked genes were classified as non-hemizygous when an apparently intact Y-linked gametolog was present [21] and as male-hemizygous otherwise. All pseudoautosomal (PAR) genes identified previously [21] were excluded from further analyses.

### 2.4. Phylogenetic Reconstruction

The phylogeny for the six *Silene* species was reconstructed using maximum likelihood (ML) in RAxML v8.0.1 [42]. A supermatrix approach was used, by concatenating all four-fold degenerate sites identified across all orthologous genes and excluding sites with more than 50% missing data. Phylogeny reconstruction employed 100 rapid bootstrap replicates [45] followed by a thorough ML search, using the General Time Reversible nucleotide substitution model (GTR) with rate heterogeneity between sites modelled with the CAT approximation (GTR+CAT). 

The extent of phylogenetic conflict among genes was assessed with the Shimodaira–Hasegawa test (SH-test) [46]. For each orthologous gene, a ML unconstrained gene tree was obtained with PhyML v3.1 [47], using the GTR+Γ model. A second phylogenetic tree was obtained for each gene using the same settings but fixing the topology to the one obtained with the supermatrix approach (using all orthologous genes). For each of these two phylogenies, individual site likelihoods reported by PhyML were used to perform the SH-test in CONSEL v1.2 [48]; all the genes for which the gene tree was significantly preferred over the species tree (FDR-corrected *p* < 0.05 [49]) were excluded from further analyses.

### 2.5. Analyses of Selection on Coding Sequences Using dN/dS Methods

For each orthologous gene confidently assigned to either X-linked or autosomal classes, the selection acting on coding sequences was analysed with methods based on the estimation of the nonsynonymous (*dN*) to synonymous (*dS*) substitution rate ratio (*ω* = *dN*/*dS*) [50], using the program Codeml from the package PAML v4.8 [51]. Two approaches were used: a pairwise estimation of *dN*/*dS* between *S. latifolia* and *S. vulgaris*, and a phylogenetic-based approach that estimates *dN*/*dS* across all six *Silene* species. For the latter, three types of models were used: “branch models” that allow ω to vary among the branches of the tree but not among the codons within genes; “sites models” that allow *ω* to vary among codons within each gene but not between the branches of the tree; “branch-sites models” allowing *ω* to vary among codons of the gene and branches of the phylogeny. 

The “branch models” used included two nested models: the one-clade model, which assumes that all branches across the phylogeny share the same *dN*/*dS* ratio, and a two-clade model, which allows different *dN*/*dS* ratios on different branches of the phylogeny. For this analysis, we defined as “foreground” branches all the branches in the dioecious clade, including *S. latifolia*, *S. dioica*, and *S. diclinis*, and as “background” branches all the other branches in the 6-species phylogeny. The relative fit of these two models to the data was compared with a likelihood ratio test (LRT) assuming χ^2^ distribution with one degree of freedom, with genes significantly preferring the two-clade model inferred to experience different selective pressures on dioecious and non- dioecious species.

The “site models” used included two pairs of nested models (M1/M2 and M7/M8 [50]). The M1 and M7 models allow for only purifying selection or neutral evolution (*ω* ≤ 1 on all codons), while the M2 and M8 models also allow for a class of codons under positive selection (with *ω* >1). The likelihood ratio tests (LRT) for both pairs of nested models were performed using χ^2^ distribution with two degrees of freedom. Genes significantly preferring the more complex models (M2, M8) were inferred to contain some codons that evolved under positive selection at some point during diversification of the six *Silene* species.

The “branch-site” analysis was performed with model A [52], which allows for “background” and “foreground” branches to have separate selective regimes. Foreground and background branches were defined as for the branch models test described above. The LRT for the “branch-site” analysis included the comparison of model A—which allows for some sites to be evolving under positive selection—to a nested simpler model where all sites are evolving either neutrally or under purifying selection (option “fix omega” in Codeml). The significance of this LRT was tested using χ^2^ distribution with one degree of freedom. Genes significantly preferring the full model A were inferred to contain some codons that evolved under neutrality or purifying selection on the background branches (non-dioecious species) and under positive selection on the foreground clade (dioecious species).

### 2.6. DNA Polymorphism Analyses

For DNA polymorphism analysis we used five female *S. latifolia* transcriptomes, four of which were published previously [21,25,30]. Trimmed raw reads of each individual were mapped to the reference female transcriptome previously published [21], with BWA v0.7 [53] with the “mem” option. Duplicate reads were removed with SAMtools v1.2 [54], and regions around indels were realigned with GATK v3.4 [55]. SNP (single-nucleotide polymorphism) calling was done for each individual plant separately, with SAMtools and BCFtools v1.2 (part of SAMtools package) using the alternative multiallelic variant caller (-m option) and including homozygous blocks with minimum depth of 8 (-g 8), after excluding reads with mapping quality below 20 (-q 20) and bases with base quality below 20 (-Q 20). SNPs with fewer than eight reads supporting them, within 3 bp of an indel, with quality below 10, or with fewer than two reads supporting each allele (for heterozygous calls) were marked as low-quality and excluded from further analysis. The resulting VCF files including confident SNP calls and homozygous blocks were converted to FASTA format using VCF2FAS [56], with heterozygous SNPs coded with IUPAC symbols. The average heterozygosity (π) and Tajima’s D [57] were calculated for silent sites for each sex-linked and autosomal gene using mstatspop [58].

### 2.7. Sex-Biased Gene Expression

To identify sex-biased genes, expression values were calculated in 23 males and 34 females for which the transcriptome data were published previously [21]. Trimmed raw reads were mapped with RSEM [59] to the published reference transcriptome of female *S. latifolia* [30], and the expression values for each gene were calculated as the number of fragments per kilobase per million reads mapped (FPKM). In this analysis, we tested only for female-biased expression because (i) the reference transcriptome we used originated from a female plant, so male-biased genes are underrepresented in that transcriptome, and (ii) according to the faster-X theory, only female-bias is expected to strongly affect the selective pressure in X-linked genes, while the effect of male-biased expression is modest at best (e.g., see Figure 1 in [9]). Female-biased genes were defined as genes with significantly (*t*-test, *p* < 0.001) higher expression in females. 

### 2.8. Analyses of Codon Bias 

To analyse codon usage bias in the dioecious and non-dioecious species, the effective number of codons (ENC, [60]) and the optimal codon frequency (FOP, [61]) for each gene were estimated with CodonW [62]. The optimal codons in *S. latifolia* were defined previously [63]. To test whether evolution of dioecy and sex chromosomes affected the selection on codon bias, the number of changes from preferred to unpreferred (P => U) synonymous codons and the number of changes in the opposite direction (U => P) were calculated between *S. latifolia* and *S. vulgaris*. To infer on which lineage these changes occurred (i.e., whether on the lineage leading *to S. vulgaris* or to on that leading to *S. latifolia*), the ancestral coding sequences of each gene were obtained with the ancestral reconstruction method implemented in PAML [64]. The modern *S. latifolia* and *S. vulgaris* sequences were compared with the sequence of their common ancestor, and the number of P => U and U => P synonymous codon changes on each lineage were counted with a custom script.

## 3. Results

### 3.1. Substitution Rates in X-Linked and Autosomal Genes

**Figure 1:**
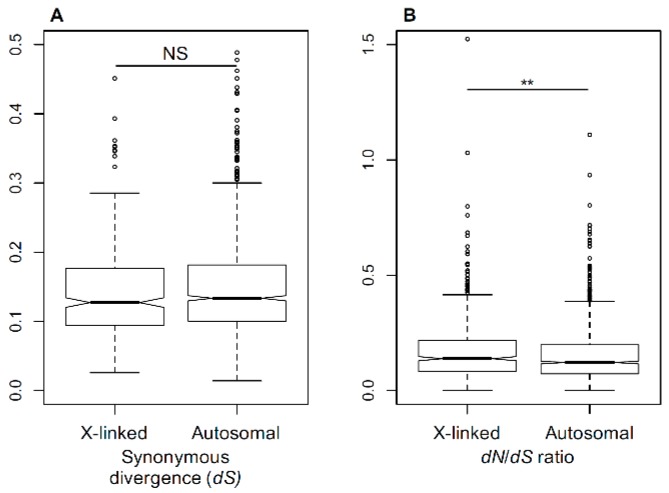
Evolutionary rates in *Silene* X-linked (X) and autosomal (A) genes. (**A**) Synonymous divergence between *Silene latifolia* and *Silene vulgaris* at X-linked and autosomal genes in *S. latifolia*. (**B**) Nonsynonymous (*dN*) to synonymous (*dS*) divergence ratio *(dN*/*dS*) between *S. latifolia* and *S. vulgaris* genes. The sex-linked genes have significantly higher *dN*/*dS* ratio than the autosomal genes (Student’s test, ** *p* < 0.01).

The rate of sequence divergence depends on the underlying mutation rate and fixation probability of the mutations. Silent mutations, such as four-fold degenerate sites at protein coding regions, are widely assumed to be neutral. Thus, a comparison of silent sequence divergence between the species for X-linked and autosomal genes allows one to test whether the mutation rates are different between the X-chromosome and the autosomes. For this purpose, we measured the silent sequence divergence (*dS*) between previously published [21,30] genes in *S. latifolia* and its non-dioecious relative *S. vulgaris*. A comparison of *dS* between 472 X-linked and 1162 autosomal genes revealed no significant difference (Student’s test, NS) (Figure 1A), indicating that the rate of synonymous divergence is similar between autosomes and sex chromosomes. The non-synonymous divergence normalised by silent divergence (*dN*/*dS*) that is often used to analyse and compare selective pressures in protein-coding regions [65] was significantly higher in X-linked than autosomal genes (Student’s test, *p* < 0.01, Figure 1B). This suggests differences in how selection works on the X-chromosome and the autosomes in this species and indicates the presence of a relatively weak, but significant, faster-X effect in *Silene*.

### 3.2. Faster-X Evolution Following Transition to Dioecy

**Figure 2:**
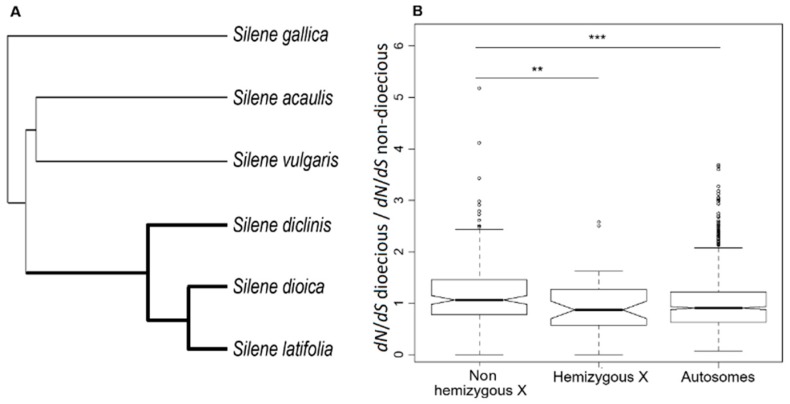
Phylogeny-based analyses of substitution rates. (**A**) The topology of the phylogenetic tree used in the analyses. All nodes had 100% bootstrap support. The “foreground” branches representing the dioecious clade are shown as thick lines. The “background” branches representing non-dioecious species are shown as thin lines. (**B**) Relative change in *dN*/*dS* ratio in the dioecious compared to non-dioecious species estimated in the branch analysis allowing separate *dN*/*dS* ratios for the “foreground” and “background” branches of the tree shown in panel A. The significance (** *p* < 0.01; *** *p* < 0.001) of the differences between the distributions of values in the panel B was tested with the Student’s test.

To reveal the causes of faster-X evolution in *S. latifolia*, we took advantage of the relatively recent origin (~10MY) of dioecy and sex chromosomes in this species that allowed us to test how the *dN*/*dS* ratios change once a gene evolves X-linkage. For this purpose, we sequenced the transcriptomes from females of three dioecious species that share the same pair of sex chromosomes (*S. latifolia*, *S. dioica,* and *S. diclinis*) and three non-dioecious *Silene* species (*S. vulgaris*, *S. gallica*, and *S. acaulis*) that effectively represent an ancestral state before separate sexes and sex chromosomes evolved in the *S. latifolia* ancestor (Figure 2A). To avoid the biases caused by mapping sequence reads to a heterospecific reference, the transcriptomes from every species were assembled de novo, and orthologous genes were identified using phylogenetic and clustering methods. Only orthologous genes containing sequences from all six species were retained for further analyses, resulting in 1129 genes present in all six species and with known linkage in *S. latifolia* [21,30], including 204 X-linked (PAR-excluded) and 925 autosomal genes. Although the linkage of the genes we analysed is known only for *S. latifolia*, we assumed the same linkage pattern in all three dioecious species and hereafter refer to orthologous genes as “X-linked” and “autosomal” for brevity. The tree topology shown in Figure 2A was used in all the phylogeny-based analyses of substitution rates described below. This topology was obtained using ML phylogenetic reconstruction in *RAxML* [42] based on the concatenation of 1,779,961 four-fold degenerate sites across 8752 orthologous genes. All the nodes in this tree had 100% bootstrap support.

For every gene, we estimated the *dN*/*dS* ratio before and after evolution of dioecy (Figure 2A), using branch models implemented in PAML [51]. The two-rate branch model allowed for separate *dN*/*dS* ratios for the “foreground” and “background” branches shown in Figure 2A as thick and thin lines, respectively. We tested whether a gene evolved under significantly different selective pressure following transition to dioecy by comparing the fit of the two rates model with the nested model, allowing for only one rate (one *dN*/*dS*) across the tree. The two-rate/one-rate LRT was significant for 29 (14.2%) X-linked and 128 (13.8%) autosomal genes (Table 1). The proportion of genes with significant LRT was not significantly different between the X-linked and autosomal genes (2×2 contingency χ^2^, *p* = 0.9). 

Using the *dN*/*dS* ratios from the two-rate model, we compared how the transition to dioecy affected the *dN*/*dS* ratios of the X-linked and autosomal genes. Little change in the *dN*/*dS* ratio was apparent for autosomal genes following transition to dioecy (Figure 2B). On the contrary, the genes that became X-linked in the dioecious clade but still retained intact Y-linked gametolog (“non-hemizygous X” in Figure 2B) showed a significant shift to higher *dN*/*dS* ratio (Student’s test, *p* value < 0.01). On the other hand, the loss of function of a Y-linked gametolog (e.g., due to premature stop codons) significantly reduced the *dN*/*dS* ratio for X-linked genes (the “hemizygous X” in Figure 2B; Student’s test, *p* value < 0.01). This indicates that haploid selection in hemizygous males significantly affects the substitution rate in *S. latifolia* X-linked genes.

### 3.3. Positive Selection in X-Linked and Autosomal Genes

To analyse whether the faster-X divergence reported above is driven by faster adaptation on the X-chromosome, we used site models (M1a, M2a, M7, and M8) implemented in PAML [51] and tested for positive selection in 204 X-linked and 925 autosomal 6-species datasets. The results of these analyses are not consistent with a more prevalent adaptive evolution in X-linked compared to autosomal genes (Table 1). In particular, the proportions of genes evolving adaptively according to the M1a/M2a and M7/M8 LRTs were lower in X-linked compared to autosomal genes (Table 1), though the difference between the X-chromosome and the autosomes was not significant (2×2 contingency χ^2^ tests), likely because of the relatively small number of genes analysed.

**Figure 3:**
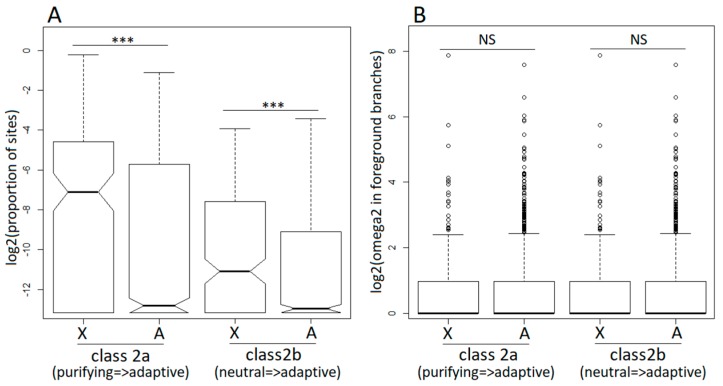
Branch-site model analysis of positive selection in the dioecious clade (foreground branches) for X-linked (X) and autosomal (A) genes. Class 2a and class 2b are classes of codons that switched to positive selection in the foreground branches from purifying selection (class 2a) and neutral evolution (class 2b) in the background branches. For the designations of foreground and background branches, see Figure 2A. (Panel **A**) shows the proportions of sites that switched to positive selection in X-linked and autosomal genes following transition to dioecy. (Panel **B**) shows the values of the omega parameter (=*dN*/*dS*) for sites that switched to positive selection in the foreground branches. The significance of the difference between the distributions was tested with the Wilcoxon rank sum test (*** *p* < 0.001).

The M1a, M2a, M7, and M8 models allow the *dN*/*dS* ratio to vary across codons but not across branches, effectively averaging across the entire tree, including the dioecious and non-dioecious species. To analyse how selective pressures change once a gene evolves sex-linkage, we employed the branch-site model A [52] implemented in PAML [51] that allowed us to test for positive selection specifically in the dioecious clade (Figure 2A). Similar to the site models described above, the branch-site model A detected adaptive evolution in a smaller proportion of X-linked compared to autosomal genes (Table 1), though this difference was not significant (2×2 contingency χ^2^ tests). Restricting the analyses to the hemizygous X-linked genes, only slightly increased the proportion of positively selected genes, though it remained lower compared to the autosomal genes (Table 1). On the other hand, the proportion of sites that became positively selected in the dioecious species was significantly higher for the X-linked compared to the autosomal genes (Wilcoxon rank sum test, *P* < 0.0001 in all X to autosome comparisons; Figure 3A). The *dN*/*dS* ratios for codons evolving adaptively in the dioecious species were not significantly different between the X-linked and the autosomal genes (Figure 3B). 

The lack of evidence for faster adaptive evolution in *Silene* X-linked compared to autosomal genes may be caused by an over-representation of female-biased genes on the *S. latifolia* X-chromosome [3] because strongly female-biased genes are not exposed to haploid selection in hemizygous males [66]. Consistent with a previous report [3], we found significant (2×2 χ^2^ = 169.63; *p* < 0.000001) enrichment of female-biased genes on the X-chromosome. However, we did not find any evidence that positive selection is less common in female-biased genes compared to unbiased genes on the X-chromosome (Table 1).

### 3.4. Purifying Selection in X-Linked and Autosomal Genes

**Figure 4:**
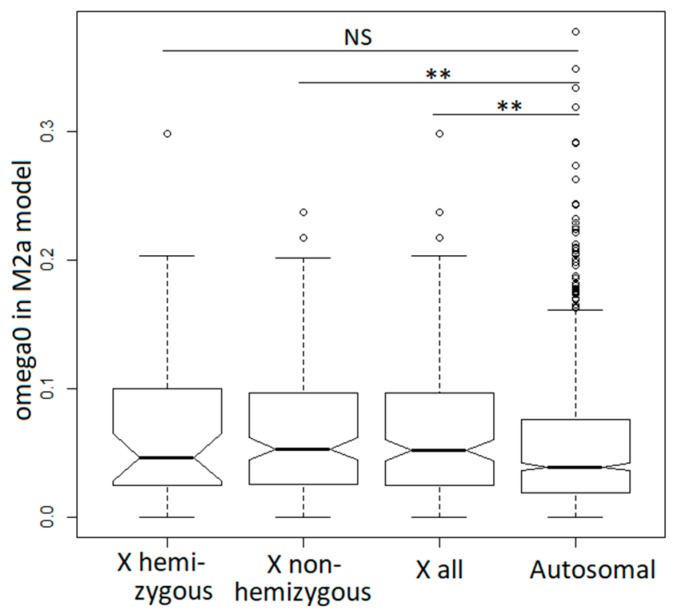
Relaxed purifying selection in X-linked compared to autosomal genes. The significance was tested with the Wilcoxon rank sum test (** *p* < 0.01).

The site model M2a described above provides separate estimates of the *dN*/*dS* ratio for codons evolving under purifying and adaptive selection [67]. This allowed us to test whether purifying selection is more stringent or more relaxed on the X-chromosome compared to the autosomes. For this purpose, we compared the estimates of the *dN*/*dS* ratio for codons evolving under purifying selection—the “ω_0_ class” in model M2a. The distribution of ω_0_ values for X-linked genes was significantly shifted upwards compared to the autosomal genes (Wilcoxon rank sum test W = 106,440, *p* = 0.0041; Figure 4). The same held true for the comparison of autosomal and non-hemizygous X-linked genes (Wilcoxon rank sum test W = 85,099, *p* = 0.0053), but not for autosomal and hemizygous X-linked genes (Wilcoxon rank sum test W = 20,415, *p* = 0.3989), likely because of the smaller number of genes in the X-hemizygous class.

### 3.5. Selection on Codon Usage Bias

**Figure 5:**
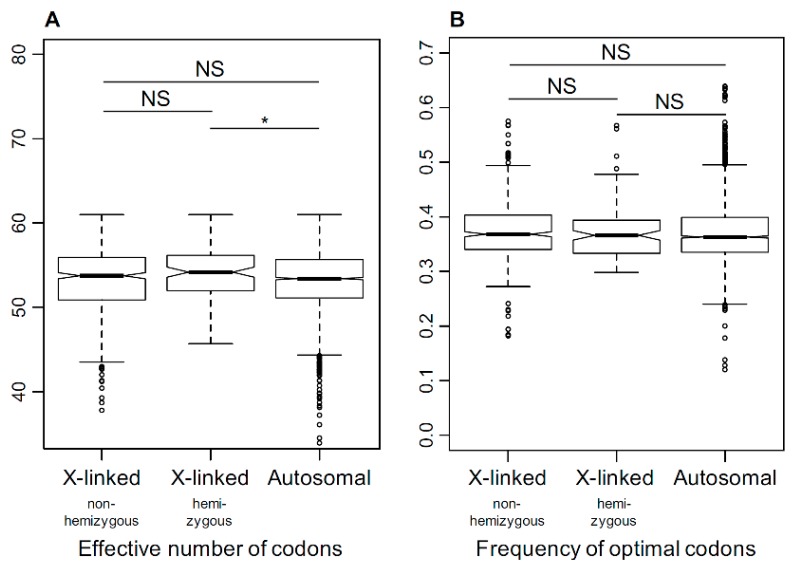
The comparison of codon bias in X-linked and autosomal genes, summarized as (**A**) effective number of codons (ENC) [60] and (**B**) frequency of optimal codons (FOP) [61]. All comparisons are non-significant (NS), except for the autosomal versus X-linked hemizygous genes for ENC, where the latter had marginally (Student’s test, * *p* = 0.0144) higher ENC (i.e., weaker codon bias).

The analysis of codon usage bias provides an alternative way to compare the strength of selection between X-linked and autosomal genes [68]. Codon bias, quantified as the effective number of codons (ENC, [60]) and the optimal codon frequency (FOP, [61]), revealed little difference between the X-linked and the autosomal genes, with the only significant difference observed between X-linked hemizygous and autosomal genes for ENC (effective number of codons) (Figure 5). Across the three dioecious species, the average ENC was 53.38, 53.98, and 53.22 for non-hemizygous X-linked, hemizygous X-linked, and autosomal genes, respectively. The ENC for the non-dioecious *Silene* averaged 53.73 and 53.3 for genes that are X-linked and autosomal in *S. latifolia*. The ENC did not differ significantly between dioecious and non-dioecious species (Student’s test, *p* = 0.58 and 0.30 for autosomal and X-linked genes). The FOP estimates, based on the 21 preferred codons defined previously for *S. latifolia* [63], did not show any differences between the two species groups (Student’s test, *p* = 0.53 and 0.52 for autosomal and X-linked genes, respectively). No significant differences were detected in either FOP or ENC between sex-linked and autosomal genes (Student’s test, ENC: *p* = 0.79, FOP: *p* = 0.44) or between X-linked hemizygous and non-hemizygous X-linked genes (Student’s test, ENC: *p* = 0.48, FOP: *p* = 0.43) in *S. latifolia*. 

Furthermore, we analysed the number of preferred to unpreferred (P => U) and unpreferred to preferred (U => P) codon changes in dioecious *S. latifolia* and non-dioecious *S. vulgaris* (Table S1) using their ancestral sequence reconstructed with the maximum likelihood approach [64]. The only significant difference detected in this analysis was a marginally higher proportion of P => U changes in the X-linked genes in the *S. latifolia* lineage compared to the homologous genes in the *S. vulgaris* lineage (2×2 Fisher’s exact test, *p* value = 0.04), suggesting a slight relaxation of selection maintaining codon bias in *S. latifolia* X-linked genes. The autosomal genes in the same comparison showed no significant difference between *S. latifolia* and *S. vulgaris* (2×2 Fisher’s exact test, *p* = 0.12). Splitting the X-linked genes into hemizygous and non-hemizygous revealed no significant differences between the groups. The comparisons between X-linked and autosomal genes within each of the *S. latifolia* and *S. vulgaris* lineages revealed no significant differences (2×2 contingency χ^2^ tests, *p* > 0.05).

### 3.6. Genetic Diversity in X-Linked and Autosomal Genes

**Figure 6:**
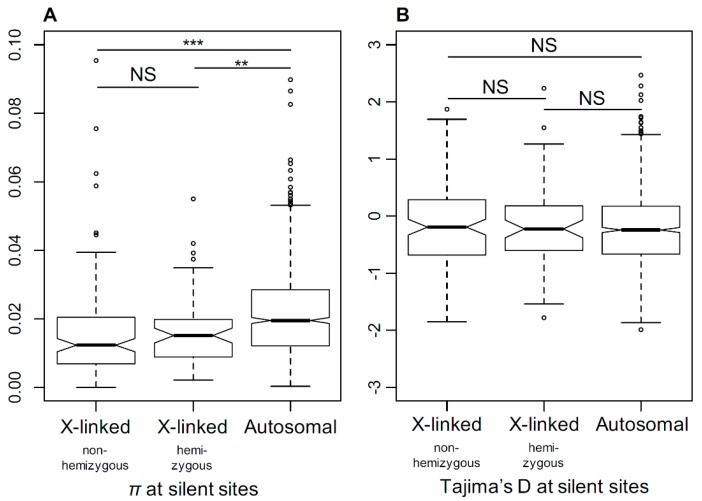
The comparison of silent genetic diversity in *S. latifolia* X-linked and autosomal genes. The comparison of silent genetic diversity (π, panel **A**) and Tajima’s D (panel **B**) for X-linked and autosomal genes. The difference in nucleotide diversity (π) between X-linked and autosomal genes is highly significant (Student’s test, *** *p* < 0.001, ** *p* < 0.01).

The relaxation of selection in X-linked genes in dioecious *Silene* species suggests a smaller *N*_e_ for the X-chromosome compared to the autosomes. To test this, we compared the genetic diversity in *S. latifolia* X-linked and autosomal genes. To estimate the genetic diversity, we used transcriptome sequence data for five *S. latifolia* females to assemble a dataset comprised of sequence alignments for 185 X-linked (PAR excluded) and 1761 autosomal genes genetically mapped to X-chromosome or autosomes in a previous study [21]. The genome-wide distribution of the average per-nucleotide heterozygosity (π) at silent sites is shown in Figure S1. The X-linked genes contain significantly less genetic diversity compared to the autosomal genes (Student’s test, *p* < 0.001) (Figure 6A). The ratio of silent genetic diversity in X-linked genes (π_x_ = 0.016) and autosomal genes (π_a_ = 0.023) was π_x_/π_a_ = 0.69, which is close to 0.75, as expected from the ploidy difference between the X-chromosome and autosomes, assuming equal sex ratios. The mean Tajima’s D was slightly negative on average for both X-linked and autosomal genes (Figure 6B). We did not find any significant difference in the distributions of Tajima’s D between the X-linked and autosomal genes (Student’s test, *p* = 0.42), suggesting that the lower diversity on the *S. latifolia* X-chromosome is unlikely to be caused by recent selective sweeps in X-linked genes. In addition, we did not detect any significant difference in π or Tajima’s D between the hemizygous and non-hemizygous X-linked genes (Student test, π: *p* = 0.58; Tajima’s D: *p* = 0.92).

## 4. Discussion

### 4.1. Faster-X Evolution in Silene

The theory predicts a faster rate of adaptation of X-linked compared to autosomal genes, assuming that beneficial mutations are, on average, partially recessive [7]. On the other hand, selection against partially recessive deleterious mutations is expected to be stronger on the X-chromosome compared to the autosomes [12]. Thus, the overall evolutionary rate of X-linked genes may be faster or slower than elsewhere in the genome, depending on whether it is dominated by adaptive or purifying selection. Faster-X divergence (i.e., higher *dN*/*dS*) was reported in many animal (but not plant) studies [8], and our finding of higher *dN*/*dS* for the divergence between *S. latifolia* and *S. vulgaris* is consistent with that literature. However, the basis for faster-X divergence in *Silene* appears to be different from those typically reported in the animal faster-X literature (e.g., [9]). In particular, we found little evidence for more frequent or stronger adaptive evolution driving faster-X in *Silene*; if anything, adaptive evolution on the *Silene* X-chromosome appears slower compared to the autosomes (Table 1), though this result is not significant. On the other hand, the purifying selection in X-linked genes appeared significantly relaxed compared to autosomal genes (Figure 4), which contradicts the expectation of stronger purifying selection against partially recessive X-linked mutations [8].

The lack of evidence for a role for adaptation in faster-X evolution in our data may reflect the relatively early stage of sex chromosome evolution in *S. latifolia*, with many sex-linked genes retaining functional X- and Y-linked gametologs. In particular, 80% of X-linked genes in our dataset had intact and apparently functional Y-linked gametologs. Such X-linked genes are non-hemizygous in males, allowing low-frequency recessive mutations to “hide” from selection in heterozygotes in the same way as on the autosomes. These non-hemizygous X-linked genes are affected by effective population size and sex-biased segregation, but not by haploid selection in males, allowing us to distinguish the effects of these factors. 

### 4.2. Genetic Diversity

A weaker purifying selection in *Silene* X-linked genes compared to autosomal genes may reflect the smaller effective population size of the X-chromosome (*N*_eX_) compared to the autosomes (*N*_eA_). Indeed, our analysis of DNA sequence polymorphism in *S. latifolia* females revealed that the average silent genetic diversity (π) in X-linked genes was about 70% of that in the autosomal genes. As genetic diversity is proportional to the product of mutation rate and effective population size, *N*_eX_/*N*_eA_ can be estimated from the ratio of genetic diversity in X-linked and autosomal genes, assuming equal mutation rates on the X-chromosome and autosomes. The latter assumption is justified by similar silent substitution rates in *S. latifolia* X- and Y-linked gametologs [30] and X-linked and autosomal genes (this study), indicating that the mutation rate is similar on the different *S. latifolia* chromosomes.

The *N*_eX_/*N*_eA_ ~ π_x_/π_a_ = 0.7 we estimated for *S. latifolia* is close to *N*_eX_/*N*_eA_ = ¾ expected from the ploidy difference between the X-chromosome and the autosomes. Although it is often assumed that because of ploidy difference the ratio of X- chromosome to autosomal effective population sizes (*N*_eX_/*N*_eA_) is close to three-quarters, this is dependent on sex ratios and on the differences in variances of male and female reproductive success, as well as on the differences in recombination rate in X-linked and autosomal genes [69]. In particular, this assumption was shown to be incorrect for many *Drosophila* species, where *N*_eX_/*N*_eA_ is close to, or even higher than 1 [70,71]. Our finding of π_x_/π_a_ = 0.7 suggests a relatively unbiased sex ratio for *S. latifolia*, which contradicts the reports that many wild populations of that species show significant sex ratio bias [72,73,74,75]. However, the extent and direction of bias in sex ratio likely varies among *S. latifolia* populations, which may result in the overall sex ratio to be close to 50:50.

### 4.3. Codon Bias

Codon bias—the unequal usage of synonymous codons in protein-coding genes—is thought to be maintained by selection-mutation-drift balance [76,77,78,79]. The analyses of codon bias have previously been used to analyse selective pressures (e.g., [80]) and compare the efficacy of purifying selection in *Drosophila* sex-linked and autosomal genes [68]. The previous analysis in *S. latifolia* based on 1608 expressed sequence fragments revealed a substantial codon bias in that species [63]. Interestingly, the set of preferred codons in *S. latifolia* is almost identical to that in *Arabidopsis thaliana* [63], suggesting that codon bias evolved under strong purifying selection ensuring little change across eudicot plants. Only 10 of the genes analysed in a previous study were sex-linked [63]. With a much larger number of sex-linked *S. latifolia* genes available now, we tested whether the strength of purifying selection maintaining codon bias differs significantly between X-linked and autosomal genes. Our analysis revealed little difference between these compartments of the genome. However, it is worth noting that codon bias is a relatively inert feature of the genome that changes slowly over time [76,78]. Thus, given the recent origin of sex chromosomes in *S. latifolia*, not enough time may have passed for the differences in the extent of codon bias to accumulate between X-linked and autosomal genes. On the other hand, the number of preferred (P) ⇔ unpreferred (U) codon substitutions should, in principle, be sensitive to relatively recent changes in selective pressure. Indeed, this analysis revealed a slightly higher proportion of P => U and a lower proportion of U => P changes in X-linked genes in *S. latifolia* compared to homologous non-sex-linked genes in *S. vulgaris* (Table S1). This finding is consistent with the relaxation of the purifying selection in *S. latifolia* X-linked genes, though this effect was only marginally significant. The lack of significant difference between X-linked and autosomal genes in *S. latifolia* indicates that this effect is weak at best.

### 4.4. Factors Affecting the Analyses

Our analyses of selection assumed that the genes that are X-linked in *S. latifolia* are also X-linked in the two other dioecious species and are not sex-linked in the non-dioecious species (Figure 2A). The sex chromosomes of *S. latifolia* are known to have evolved from a single pair of chromosomes in the non-dioecious ancestor [81]. The karyotype of dioecious *S. dioica* is identical to that of *S. latifolia* [82]. The third dioecious species analysed, *S. diclinis*, shares the sex chromosomes with *S. latifolia* and *S. dioica* but, in addition, it evolved an extra pair of X- and Y-chromosomes—the neo-sex-chromosomes—due to a translocation involving the Y-chromosome and an autosome [83]. Despite this translocation, the female karyotype of *S. diclinis* is indistinguishable from those of *S. latifolia* and *S. dioica* [83]; thus, we reason that it is safe to assume that the genes that were known to be X-linked in *S. latifolia* [21,30] are also X-linked in the two other dioecious species. This assumption is reasonable given that the three dioecious species are closely related, and there is a high degree of synteny in the genus, revealed by the genetic mapping of many genes in dioecious *S. latifolia* and non-dioecious *S. vulgaris* [81,84].

Our phylogeny-based analyses compared selective pressures between the dioecious and non-dioecious species, which implicitly assumed that all the X-linked genes have become X-linked immediately following divergence from the non-dioecious ancestor. This is likely an unrealistic assumption, as dioecy and sex chromosomes may have taken some time to evolve in the dioecious clade. If cessation of recombination between *S. latifolia* sex chromosomes occurred in several steps, as evidenced by the variation in silent divergence between homologous X- and Y-linked copies [28,85], then different X-linked genes became sex-linked at different times in the past. Furthermore, as Y-degeneration is a stochastic process [1], different X-linked genes lost functional Y-linked gametologs at different time since the gene becomes sex-linked. Thus, at least some of the X-linked hemizygous genes may have lost Y-linked gametologs very recently, which reduced the power of the phylogeny-based analyses to detect the change in selective pressure caused by the exposure to haploid selection in males. Furthermore, the presence of intact Y-linked gametologs for X-linked genes was analysed only in *S. latifolia* [21,30], meaning that in the other two dioecious species some X-hemizygous genes may not be X-hemizygous, which may have blurred the distinction between these groups of X-linked genes. However, it is worth noting that the three dioecious species used in this study are very closely related, so the differences between their sex-linked genes are likely to be minimal.

The set of genes we used in the analyses was limited by two factors. Firstly, we required a gene to be present in each of the de novo-assembled transcriptomes of the six *Silene* species analysed. Secondly, we focused the analysis on the genes that were previously genetically mapped to the X-chromosome or the autosomes [21]. This significantly reduced the number of genes available for the analyses and likely biased the selection of genes towards constitutively and actively expressed genes. This bias is unlikely to have affected the X-linked and autosomal genes differently, so the X/A comparisons of genetic diversity and selective pressures should not be affected. Another likely bias in the set of genes we analysed is the under-representation of male-biased genes due to the use of female-transcriptomes in the dioecious *Silene* species. However, this is unlikely to undermine the conclusions of the study because only female-bias is expected to strongly affect the selective pressure in X-linked genes, while the effect of male-biased expression is relatively weak (e.g., see Figure 1 in [9]). Despite the expected strong dependence of faster-X effect on female bias, we detected no difference in the prevalence of adaptive evolution between female-biased and unbiased X-linked genes. This result may be attributed to a lack of power due to the relatively small number of X-linked genes analysed.

The power and reliability of the phylogeny-based analyses of selection depend on the assumed phylogenetic history of the species analysed [86,87]. The phylogeny we used for the analysis (Figure 2A) is based on 8752 genes—far larger than in previous phylogenetic studies in this genus (e.g., [88]). Our six-species phylogeny was highly supported in bootstrap analyses and fitted well with the recognised species relationships in the genus *Silene* [88,89]. Furthermore, all the analyses shared the same underlying tree topology, and any potential problems with the phylogeny should have equally affected X-linked and autosomal genes. Thus, it is unlikely that problems with the underlying species phylogeny could have biased the relative proportions of X-linked and autosomal genes evolving under positive and purifying selection.

## 5. Conclusions

Our results indicate that the evolution of X-linked genes in *S. latifolia* and its dioecious relatives is dominated by relaxation of selection due to a smaller effective population size of the X-chromosome compared to the autosomes. This conclusion is consistent with lower genetic diversity and higher *dN*/*dS* ratio in X-linked compared to autosomal genes. Furthermore, our phylogeny-based analyses of selection reveal weaker purifying selection and lack of faster adaptation in X-linked genes. We hypothesize that the lack of faster-X adaptation in *Silene* is due to the presence of intact Y-linked gametologs for most X-linked genes analysed. This non-hemizygosity of X-linked genes may have prevented the exposure of partly recessive mutations to haploid selection in males that is thought to be the driver of faster-X adaptation in older sex chromosomes, as found in mammals and *Drosophila*, where all or nearly all X-linked genes are hemizygous in males. The lack of faster-X adaptation and the partial relaxation of purifying selection may well be general features of recently evolved sex chromosomes with non-degenerate or partially degenerate Y-chromosomes.

## Figures and Tables

**Table 1:** Likelihood ratio tests (LRT) for adaptive evolution in six-species datasets.

		df	LRT at 5% Significance						LRT at 1% Significance				
	Model	in	X-Linked (PAR Excluded)				Autosomal		X-Linked (PAR Excluded)				Autosomal
	Type	LRT	All	Hemizygous	F-Biased		All		All	Hemizygous	F-Biased		All
Genes:			204	41	84		925		204	41	84		925
2 rate/1 rate	branch	2	18(8.8%)	5(12.2%)	7(8.3%)		73(7.9%)		11(5.4%)	1(2.4%)	4(4.8%)		55(5.9%)
M2a/M1a	site	2	12(5.9%)	3(7.3%)	5(5.95%)		84(9.1%)		5(2.5%)	1(2.4%)	2(2.4%)		49(5.3%)
M8/M7	site	2	24(11.8%)	5(12.2%)	8(9.5%)		145(15.7%)		7(3.4%)	2(4.9%)	4(4.8%)		82(8.9%)
new model A	br.-site	1	6(2.9%)	1(2.4%)	3(3.6%)		55(5.9%)		2(0.98%)	1(2.4%)	0		27(2.9%)

PAR: pseudoautosomal region; F-biased: female-biased; LRT: likelihood ratio tests.

## Supplementary Materials

The following are available online at http://www.mdpi.com/2073-4425/9/5/234/s1. Figure S1: Genetic diversity at silent sites of X-linked and autosomal genes, Table S1: The numbers of preferred (P) and un-preferred (U) codon changes.
