# Supplementary material for: A Comparison of Selective Pressures in Plant X-Linked and Autosomal Genes
Source: Genes (Basel). 2018 May 3;9(5):234. doi: 10.3390/genes9050234 (PMC5977174; doi:10.3390/genes9050234)
Supplement: Supplementary file 1 [file genes-09-00234-s001.pdf]

Supplementary Materials:

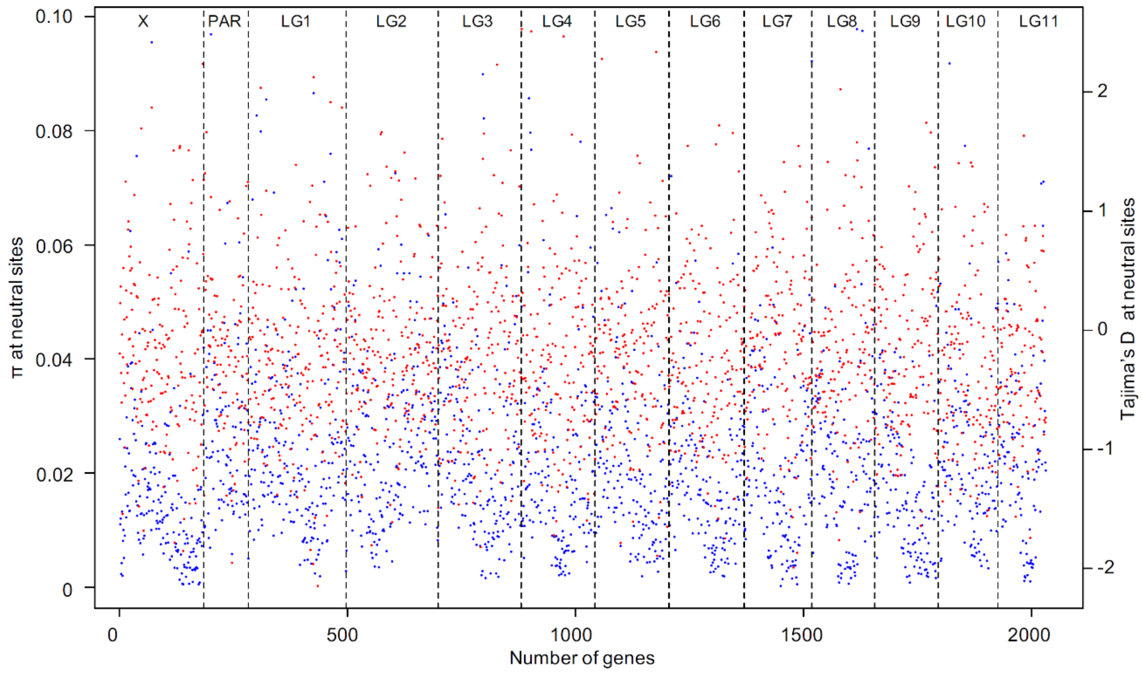

Supplementary figure S1. Genetic diversity at silent sites of X-linked and autosomal genes mapped in the previous study [21]. The per nucleotide average heterozygosity (π) values are shown in blue; the Tajima's D values are in red.

Supplementary table S1. The numbers of preferred (P) and un-preferred (U) codon changes in *S. latifolia* and *S. vulgaris* lineages from their common ancestor, assuming preferred codon set defined in the previous study [56].

	S. latifolia		*S. vulgaris*	
	**X**	**A**	**homologous to X-linked**	**homologous to autosomal**
**P=>U**	510	1721	575	1748
**U=>P**	295	870	276	949
**total**	805	2591	851	2697
